# Intravenous Infusion of Autologous Plasma and Buffy Coat Components Enhances Natural Killer Cell Activity: A Case Report

**DOI:** 10.7759/cureus.105873

**Published:** 2026-03-26

**Authors:** Yongho Lee

**Affiliations:** 1 Pain Management, LeadM Pain Clinic, Daegu, KOR

**Keywords:** anti-aging medicine, autologous blood injection, buffy coat, immune enhancement, intravenous infusion, nk cell activity, preventive medicine

## Abstract

Immune enhancement is increasingly recognized as an important goal not only for patients with chronic illnesses but also for healthy individuals seeking preventive health. Natural killer (NK) cell activity serves as a sensitive biomarker of immune competence, and subclinical deficits may exist even in asymptomatic, otherwise healthy adults. We report a case of a young, female, asymptomatic adult with no underlying disease, in whom markedly decreased NK cell activity was incidentally detected during routine immune function assessment. She had a history of persistent leukopenia, prompting further laboratory testing despite being apparently healthy. No clinical symptoms were present. The patient received intravenous autologous blood therapy, including plasma and buffy coat components, at regular intervals. Serial laboratory monitoring revealed a dramatic and progressive increase in NK cell activity, from a subclinical baseline to well above the normal reference range. Leukocyte and platelet counts remained stable, and no adverse events or subjective symptoms were observed. This case suggests that intravenous autologous blood therapy may enhance NK cell activity in selected individuals with subclinical immune deficits. However, the clinical significance and potential broader applications, including preventive or anti-aging benefits, remain uncertain and require further investigation.

## Introduction

In recent years, enhancement of immune function has become a focus not only for patients with chronic illnesses but also for asymptomatic individuals seeking to maintain optimal health and prevent disease. Immune cells such as leukocytes and natural killer (NK) cells are essential components of innate immunity and serve critical roles in immune surveillance [1].

Leukocytes are white blood cells responsible for defending the body against infections and foreign substances. Natural killer (NK) cells are a subset of lymphocytes that mediate spontaneous cytotoxicity against virus-infected and malignant cells without prior sensitization [1,2]. Accordingly, NK cell activity has been established as a biomarker of immune competence and is increasingly used in both clinical and research settings to assess immune function [2]. Regular assessment of immune cell function, including NK cell activity, is increasingly available in populations at risk for immune suppression and is also being incorporated into preventive health strategies in otherwise healthy individuals [2].

The clinical significance of low NK cell activity in asymptomatic individuals is not fully established. Still, subclinical deficits may be associated with increased susceptibility to infections and malignancy, even in the absence of overt symptoms. Thus, identifying and addressing such immune deficiencies may be relevant for preventive health, although further research is needed to clarify long-term outcomes.

Current immunotherapy recommendations and most clinical applications primarily address patients with overt disease, such as cancer or autoimmune conditions, in which immune modulation is central to disease management. Meanwhile, there is growing interest in proactive immune enhancement in asymptomatic individuals, driven by an aging society, environmental stressors, and increasing public awareness of preventive medicine [1,3]. While some claim potential “anti-aging” benefits from immune enhancement, it is important to frame such claims cautiously and acknowledge that robust clinical evidence supporting anti-aging effects remains limited.

Autologous blood-derived therapies, including platelet-rich plasma (PRP) and autologous plasma-based interventions, have been extensively studied for their tissue regeneration and immunomodulatory properties [4-7]. Platelet-rich plasma is a blood product enriched with platelets, growth factors, and cytokines, prepared by centrifuging whole blood. The buffy coat is the cellular fraction of blood obtained after centrifugation, which is enriched with leukocytes and platelets, and is considered to have immune-stimulating properties through its concentrated population of immune cells [6]. However, clinical evidence regarding the effect of these approaches on NK cell activity, particularly when administered as intravenous infusions in healthy, asymptomatic adults, remains limited.

This report presents a case of a healthy adult with no symptoms or underlying disease in whom markedly decreased NK cell activity was incidentally identified during routine immune function assessment. Following intravenous infusion of autologous plasma and buffy coat components, NK cell activity increased substantially and objectively on serial testing. This observation demonstrates an association between autologous blood-derived infusions and increased NK cell activity in a single asymptomatic adult with subclinical immune deficiency. The clinical significance of this finding remains uncertain, and further research is required to determine whether such interventions have a role in preventive medicine or immune support.

NK cell activity, commonly measured via interferon-gamma (IFN-γ) release assays, is considered a clinically relevant indicator of innate immune function [2]. Decreased NK cell activity has been associated with increased susceptibility to infections and malignancy, whereas restoration of NK cell function may be beneficial [2,5]. Accordingly, serial NK cell activity measurements before and after intervention may provide a practical framework for immune monitoring in preventive medicine and for evaluating immune-enhancing interventions.

## Case presentation

Case presentation

A 39-year-old female with no underlying disease or symptoms underwent routine laboratory testing as part of a preventive health assessment. Notably, she had a history of persistent leukopenia for several years, for which she had previously received evaluation at a tertiary university hospital. Comprehensive diagnostic workup at that time, including differential blood counts, autoimmune panel, infection screening, and bone marrow assessment, revealed no abnormal findings, and she was advised to continue observation without specific treatment.

At the time of presentation to our clinic, laboratory evaluation again showed leukopenia with a white blood cell (WBC) count of 2.61 ×10³/μL (reference range: 4.0-10.0 ×10³/μL) and markedly reduced NK cell activity of 77 pg/mL (reference range: >500 pg/mL), despite the absence of any clinical symptoms or signs. The patient reported no history of prior infections, fatigue, weight loss, autoimmune disease, medication use, recent vaccinations, or environmental exposures that could potentially explain the laboratory abnormalities. Physical examination was unremarkable, and no clinical symptoms or signs were present at any point during evaluation and treatment.

All procedures were performed in the outpatient clinic setting at LeadM Pain Clinic, Daegu, Republic of Korea. Peripheral blood was drawn from the antecubital vein under aseptic conditions by trained medical staff. For autologous infusion, 120 mL of peripheral blood was collected at each session, anticoagulated with sodium citrate, and centrifuged at 2,800 rpm for 5 minutes using a sterile in-line filter. The separated plasma and buffy coat were mixed with 200 mL of normal saline (as intravenous fluid) and administered via intravenous infusion through the antecubital vein over 30 minutes in a dedicated procedure room, with continuous monitoring for adverse events.

This procedure was performed a total of six times at intervals of approximately 4-6 weeks. The patient was monitored for adverse events throughout the 6-month treatment and follow-up period, and no side effects, including infection, fever, or other complications, were observed. During the treatment period, the patient reported no intercurrent infections, vaccinations, new medications, or supplement use.

At each visit, routine blood tests, including WBC count, platelet count, and lymphocyte percentage, were obtained concurrently with NK cell activity testing measured by IFN-γ release assay (pg/mL). Following the initial infusion session, the WBC count demonstrated a modest increase from the baseline leukopenic level and subsequently stabilized within a relatively narrow range (3.68-4.11 ×10³/μL) throughout the follow-up period. Platelet and lymphocyte counts exhibited expected physiological fluctuations but remained within clinically acceptable parameters without demonstrating any progressive abnormal trend.

**Figure 1:**
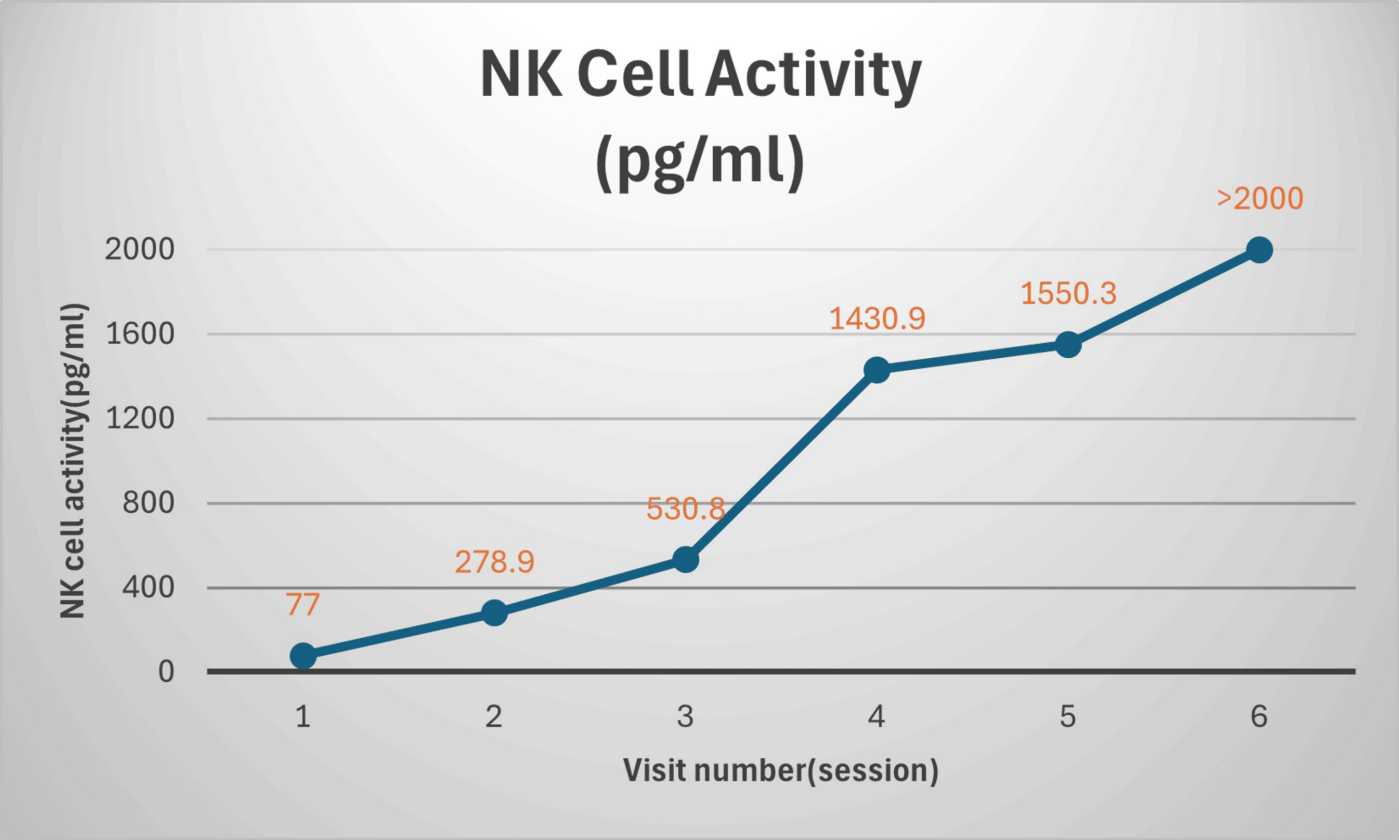
Serial changes in NK cell activity (pg/mL) Serial changes in natural killer (NK) cell activity (pg/mL) measured by interferon-gamma (IFN-γ) release assay across six sessions of intravenous autologous plasma and buffy coat infusion. NK cell activity increased markedly from a baseline of 77 pg/mL to over 2,000 pg/mL after six sessions. The reference range for NK cell activity, as reported by the testing laboratory, is ≥500 pg/mL. Values reported as “>2,000” indicate the upper limit of quantification for the assay.

In striking contrast to the stability of other laboratory parameters, NK cell activity demonstrated a dramatic and progressive increase across the six treatment sessions. The patient's baseline NK cell activity of 77 pg/mL, which represented a marked subclinical deficiency, rose substantially after the second treatment session to 278.9 pg/mL. This upward trajectory accelerated at the third session, reaching 530.8 pg/mL and crossing the threshold of the normal reference range (≥500 pg/mL). Subsequent infusions resulted in continued escalation of NK cell activity, with measurements of 1,430.9 pg/mL following the fourth session and 1,550.3 pg/mL after the fifth session. By the sixth and final treatment session, NK cell activity had exceeded 2,000 pg/mL, representing a more than 25-fold increase from baseline. These serial laboratory findings and progressive changes in NK cell activity are presented graphically in Figure 1, with corresponding numerical data provided in Table 1.

**Table 1:** Serial laboratory findings and NK Cell Activity All laboratory values were measured immediately before each infusion session. Serial measurements of white blood cell (WBC), platelet count, lymphocyte percentage, and natural killer (NK) cell activity (pg/mL) are shown. NK cell activity was determined by interferon-gamma (IFN-γ) release assay. Reference ranges: WBC 4.0–10.0 ×10³/μL, platelets 150–370 ×10³/μL, lymphocytes 20–50%. The NK cell activity reference threshold was ≥500 pg/mL. *Values reported as “>2,000” represent the upper limit of quantification for the assay.

Visit	Date	WBC (10³/μL)	Platelet (10³/μL)		Lymphocyte (%)		NK Cell Activity(pg/ml)	
									1	2025	2.61	134		33.7		77	
10-Apr																	
2	2025	4.11	96		29.9		278.9	
	23-May							
3	2025	4.41	138		19		530.8	
	23-Jun							
4	2025	3.45	128		33.3		1430.9	
	24-Jul							
5	2025	3.64	145		31.8		1550.3	
	28-Aug							
6	2025	3.68	145		29.1		>2000*	
	25-Sep							

Throughout the treatment and follow-up period, no adverse events or clinical symptoms were observed, and the patient did not report subjective improvements in fatigue or general condition despite the marked laboratory changes.

## Discussion

This case describes a clinically notable observation in which a healthy, asymptomatic adult with incidentally identified subclinical NK cell deficiency demonstrated a substantial and progressive increase in NK cell activity after intravenous infusion of autologous plasma and buffy coat components. Autologous blood-derived therapies such as PRP and plasma-based interventions have recognized roles in tissue repair and immunomodulation, but the immune effects of direct intravenous administration of plasma and buffy coat components remain comparatively underexplored, particularly in individuals without overt disease [5-8]. In the present case, leukocyte and platelet counts remained relatively stable while NK cell activity increased from an immunodeficient baseline (77 pg/mL) to levels exceeding the usual clinical threshold (>2,000 pg/mL). The absence of adverse events in this case is also notable and supports preliminary feasibility from a safety standpoint.

Assay variability was considered minimal based on laboratory standards, but alternative explanations, such as spontaneous recovery or laboratory error, cannot be fully excluded. Potential confounders, including intercurrent infections, vaccinations, medications, and supplement use, were systematically assessed and excluded during the treatment and follow-up period.

Natural killer cells play a central role in innate immunity and contribute to immune surveillance against infection and malignancy [1,2]. In clinical settings, NK cell activity is increasingly used as a practical biomarker of immune competence, and measurement of NK cell function through IFN-γ release provides an objective assessment of immune status [2]. From a preventive medicine perspective, enhancement of NK cell function in individuals with subclinical deficits could plausibly improve immune surveillance and resilience, although clinical endpoints were not assessed in this report. Despite marked laboratory changes, the patient remained asymptomatic, and the clinical significance of increased NK cell activity remains uncertain.

A notable observation from clinical practice suggests that baseline immune status may influence responsiveness to autologous blood-derived interventions. Specifically, asymptomatic individuals with normal baseline NK cell activity have not consistently demonstrated the same magnitude of increase in NK cell activity after similar interventions, whereas patients with subclinical NK cell deficiency, such as the patient presented in this case, have shown remarkable responses. This observation suggests that immune enhancement strategies may be most effective in selected individuals with demonstrated subclinical immune deficits, although this hypothesis requires systematic study and cannot be generalized based on a single case report. Claims regarding immune support or anti-aging effects should be interpreted cautiously, as this report describes a single uncontrolled case without clinical endpoints, controls, or mechanistic evidence.

Compared with ex vivo immune cell expansion and reinfusion strategies, which may entail substantial complexity, cost, and procedural risk, infusion of autologous plasma and buffy coat components may represent a simpler approach for selected individuals. As interest in preventive medicine and anti-aging interventions grows, further research is warranted to clarify the long-term effects, optimal protocols, patient selection criteria, and comparative effectiveness of autologous blood-derived infusions versus alternative immune-enhancing approaches [3,9].

Limitations

This report is limited by its single-case design, the absence of a control group, and short-term follow-up. The patient's baseline NK cell activity of 77 pg/mL represented a marked subclinical deficiency [10]. The findings may not be generalizable to all healthy individuals, particularly those with normal baseline immune function. While long-term epidemiological studies have linked reduced NK cell activity to increased cancer risk [11], the clinical significance of correcting subclinical deficiency in asymptomatic individuals remains unclear. Furthermore, the mechanisms underlying the dramatic increase in NK cell activity following autologous blood infusion warrant further investigation, particularly in the context of immunosenescence [12]. To confirm these findings and to clarify which patient populations may benefit from autologous blood-derived immune enhancement strategies, future research should include larger, well-controlled studies.

## Conclusions

Intravenous autologous infusion of plasma and buffy coat components was associated with a marked increase in NK cell activity in a healthy, asymptomatic adult with subclinical immune deficiency, without observed adverse events in this case. However, as this report describes a single uncontrolled case without clinical outcomes, no conclusions regarding clinical benefit or potential application for immune support or preventive medicine can be drawn. Further research is required to determine the clinical significance, efficacy, and safety of this approach.
